# Sensorimotor mismatch disrupts motor automaticity and increases anxiety during a goal-directed balance task

**DOI:** 10.3389/fnhum.2025.1632265

**Published:** 2025-09-25

**Authors:** Anke Hua, Kelly P. Westlake, Cédrick T. Bonnet, Jian Wang

**Affiliations:** ^1^Department of Physical Therapy and Rehabilitation Science, University of Maryland School of Medicine, Baltimore, MD, United States; ^2^Department of Sports Science, Zhejiang University, Hangzhou, Zhejiang, China; ^3^Univ. Lille, CNRS, UMR 9193 - SCALab - Sciences Cognitives et Sciences Affectives, Lille, France; ^4^Center for Psychological Science, Zhejiang University, Hangzhou, Zhejiang, China

**Keywords:** motor control, state anxiety, automaticity, conscious control, error augmentation

## Abstract

**Introduction:**

Sensorimotor integration is crucial role for goal-directed tasks, with sensorimotor mismatch impairing movement execution and potentially evoking anxiety. However, the relationship between mismatch-induced anxiety, movement precision, and automaticity remains unexplored. This study investigated the effect of sensorimotor mismatch on voluntary postural control during goal-directed tasks and the relationship between sensorimotor mismatch-induced anxiety and motor performance.

**Methods:**

Twenty-three young, injury-free adults performed a precision task requiring center of pressure (COP) control within a limited screen area under congruent (aligned visual inputs and motor outputs) and incongruent (180-degree mismatch between visual feedback and motor actions) conditions. Self-reported anxiety was assessed using a seven-point Likert scale. Motor performance was quantified using COP area, total path length and sample entropy of COP trajectory for movement precision and automaticity.

**Results:**

Sensorimotor mismatch significantly increased self-reported anxiety (*p* = 0.02) and reduced movement automaticity, evidenced by lower sample entropy values (*p* < 0.01). Higher anxiety scores were correlated with decreased movement automaticity in the medio-lateral direction (lower sample entropy) under the mismatch condition (*r* = −0.33, *p* = 0.008).

**Discussion:**

These findings suggest that sensorimotor mismatch induces self-perceived anxiety and disrupts automatic motor control processes.

## 1 Introduction

Precise and coordinated movements are essential for completing goal-directed tasks, requiring the integration of multiple sensory inputs (i.e., visual, somatosensory, vestibular) that are compared to prediction generated by internal representations (Kawato, 1999). This process optimizes motor control by minimizing effort while ensuring sensorimotor flexibility, as revealed by nonlinear dynamics (Pratviel et al., 2021). Sensorimotor mismatch, defined as incongruent feedback between sensory and motor systems, disrupts this integration by producing discrepancies in motor responses, thereby amplifying errors and impairing movement performance (Hadjiosif et al., 2021; Holmes et al., 2004).

Sensorimotor mismatch shares similarities with error augmentation strategies used in motor learning, as both approaches create discrepancies between expected and actual sensory feedback, challenging the motor system to adapt. Fasola et al. (2019) found that an appropriate error augmentation enhanced motor adaptation, whereas a larger error augmentation reduced adaptation during a goal-directed balance task. However, sensorimotor mismatch can elicit erroneous perceptions, leading to anxiety and subsequent control problems (Viaud-Delmon et al., 2011). This anxiety-motor interaction aligns with the Yerkes-Dodson principle that moderate anxiety might enhance error awareness, potentially benefiting learning, while excessive anxiety could disrupt predictive coding and impair performance. Thus, anxiety induced by sensorimotor mismatch could functionally resemble maladaptive error amplification when it exceeds an individual’s challenge threshold.

The relationship between anxiety and motor control can be explained through established theoretical frameworks. According to execution focus models, anxiety increases mental effort and conscious movement monitoring, which disrupts automatic control processes (Beilock and Carr, 2001; Nieuwenhuys and Oudejans, 2012). The constrained action hypothesis further suggests that anxiety-induced internal focus impairs the unconscious, reflexive control processes that normally regulate coordinated movement (Wulf et al., 2001). These theoretical perspectives suggest that sensorimotor mismatch-induced anxiety can lower automaticity.

While previous research has separately examined the effects of sensorimotor mismatch and anxiety on impaired motor performance (Cleworth et al., 2018; Holmes et al., 2004; Young et al., 2012), significant gaps exist in understanding their combined effects on movement automaticity and precise motor control. Specifically, the relationship between sensorimotor mismatch-induced anxiety and the automaticity of voluntary control during goal-directed tasks remains uninvestigated. This gap is particularly relevant for clinical populations with sensorimotor integration deficits, such as patients with dystonia, stroke, or cerebellar disorders, where anxiety might compound existing motor control challenges (Desrochers et al., 2019; Edwards et al., 2019; McClelland and Lin, 2021).

In our present study, we asked participants to complete a goal-directed voluntary control task under either a normal congruent condition (i.e., aligned visual inputs and motor outputs) or an incongruent condition (i.e., a mismatch between visual inputs and motor outputs). Our objective was to manipulate self-perceived anxiety via sensorimotor mismatch and to test significant relationships between mismatch-induced anxiety and voluntary motor control. We predicted that compared to the congruent condition, sensorimotor mismatch would decrease movement automaticity (lower sample entropy) and precision (larger total path length and movement area), with these impairments in motor performance being associated with increased state anxiety. These findings could have important implications for understanding how anxiety and altered sensory feedback interact to affect motor learning and performance in both healthy individuals and clinical populations.

## 2 Materials and methods

### 2.1 Participants

Based on the study showing sensorimotor mismatch reduced the accuracy of motor control and impaired the motor perception (Salomon et al., 2016), we estimated the sample size in G*Power software (version 3.1). With an estimated effect size of 0.6, a two-tailed test, an α of 0.05, and a power of 0.8, the minimum required sample size was calculated to be twenty-two. In our present study, twenty-three healthy young adults (7 females, 16 males) were recruited from Zhejiang University to participate in this study. Participants had a mean age of 25.26 ± 2.07 years, a mean weight of 62.80 ± 12.03 kg, and a mean height of 171.17 ± 12.03 cm. Healthy adults aged 18–30 were included in this study. Potential participants were excluded if they reported any musculoskeletal injuries, neurological conditions, anxiety disorder or visual impairments that could affect balance or motor control. All participants provided written informed consent before participating. The study protocol was approved by the Ethics Committee of Zhejiang University Psychological Science Research Center (2022-003).

### 2.2 Apparatus

**FIGURE 1:**
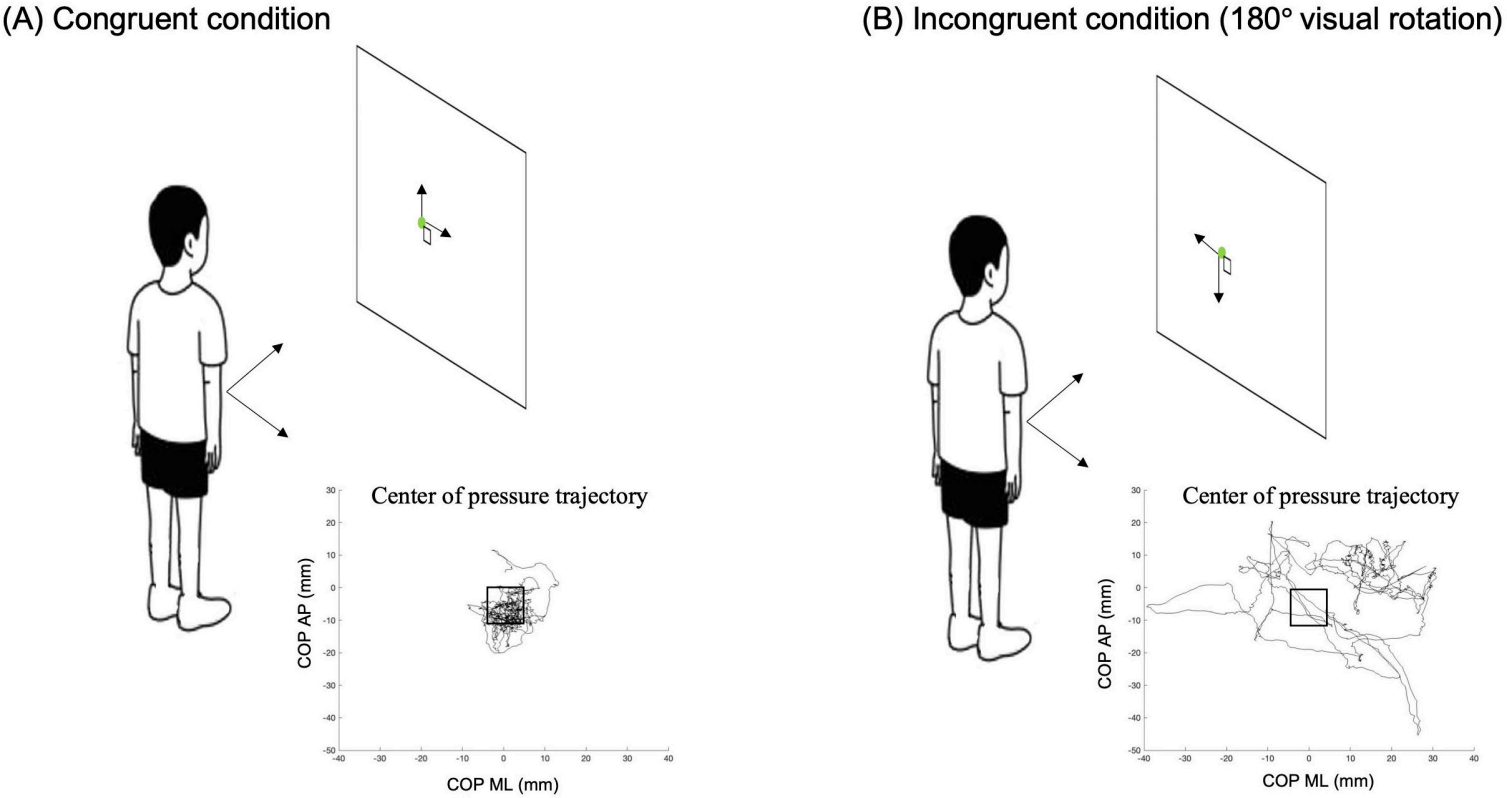
Illustration of experimental setups. **(A)** Congruent condition: participants moved their body forward or to the right, the green cursor moved up and to the right, respectively. **(B)** Incongruent condition: participants moved their body forward or to the right, the green cursor moved down and to the left, respectively.

Center of pressure (COP) data were collected using a Wii balance board (Nintendo, Kyoto, Japan) interfaced with BrainBlox software (Cooper et al., 2014) at a sampling frequency of 100 Hz. The Wii balance board was positioned 80 cm in front of a computer monitor (58.9 cm height × 33.1 cm width). The center of monitor was positioned at eye level for each participant. Within the BrainBlox software interface, participants viewed a 1 cm × 1 cm white box at the center of the screen, which served as the target area. We selected a 1 cm × 1 cm target area because it approximates the typical range of COP sway observed during quiet standing without perturbations (Ivanenko and Gurfinkel, 2018). A small green round cursor (0.5 cm diameter) displayed the participant’s real time center of pressure (COP) position (Figure 1), providing real-time visual feedback of their voluntary postural sway movements. For the congruent condition (Figure 1A), forward and leftward body movements resulted in upward and leftward cursor movements, respectively. For the incongruent condition (Figure 1B), the Wii balance board was rotated 180 °C. As a result, forward or leftward body movement resulted in downward and rightward curser movements, creating a reversed visual feedback relative to the participant’s movements.

### 2.3 Procedure

Upon arrival, each participant received detailed instructions about the experimental tasks. Participants were asked to remove their shoes and socks and stand barefoot on the Wii balance board, adopting a standardized foot position based on established guidelines (McIlroy and Maki, 1997). Before formal data collection, participants completed a familiarization period under the congruent condition. This involved actively exploring their balance limits by intentionally swaying their body forward, backward, left, and right. This step was designed to allow participants to become accustomed to the mapping between their movements and the visual feedback. Following familiarization, participants were instructed to maintain the green cursor on the screen, within the white box on the screen, as accurately as possible by controlling their body sway. Participants began each trial within the target area. The experiment consisted of three 30-s trials of the congruent task, followed by three 30-s trials of the incongruent task, presented sequentially. This ordering was intended to allow participants to establish the sensorimotor mapping in the congruent condition before exposure to the incongruent condition. We restricted the number of repetitions to three trials per condition to avoid potential short-term adaptation to sensorimotor mismatch. Each trial was followed by a 30-s rest period, during which participants rated their subjective state anxiety using a seven-point Likert scale (Meuter et al., 2003). Specifically, participants responded to the question: “How anxious do you feel when completing the task?” (1 = Not at all, 7 = Very much). The seven-point Likert scale has been used to evaluate self-reported stress, arousal, and concentration during sensorimotor control tasks (Furuya et al., 2021; Lamp et al., 2022), as well as to assess technology anxiety (Meuter et al., 2003), though it is important to note that the seven-point Likert scale is intended to capture participants’ subjective and self-perceived anxiety in our study.

### 2.4 Data analysis

No trials were excluded after confirming the absence of missing data or COP signal artifacts. COP data from each trial were processed offline using a custom script in MATLAB (R2021a, MathWorks, USA). The initial 2 s from each trial were discarded to minimize the influence of transient postural adjustments at the beginning of the trial. The remaining 28 s COP time series were then filtered using a 20 Hz low-pass, 2*^nd^* order, zero-lag Butterworth filter (Hao et al., 2021). The mean value of the filtered data was subtracted from the time series to center the COP data around zero. Both linear and nonlinear analyses were performed on the filtered COP data. COP outcome measures included total path length, COP area, and sample entropy. COP total path length was calculated using Equation 1. COP area quantified 95% of the total area using a confidence ellipse area to fit the COP data in ML and AP directions. COP total path length and area were used to quantify the precision of control. Sample entropy in the anterior-posterior (AP) and medial-lateral (ML) directions was calculated to quantify the automaticity of control (Equation 2). Lower sample entropy values have been shown to indicate reduced movement automaticity and greater conscious control (Kal et al., 2013; Kędziorek and Błażkiewicz, 2020; Richer and Lajoie, 2020; Roerdink et al., 2011).


(1) $$T\,o\,t\,a\,l\,p\,a\,t\,h\,l\,e\,n\,g\,t\,h=\overset{N}{\underset{i=1}{\sum }}\sqrt{{\left(x_{i+1}-x_{i}\right)}^{2}+{\left(y_{i+1}-y_{i}\right)}^{2}}$$

where *x_i_* and *y_i_* represent the COP coordinates in the ML or AP direction, and *N* is the number of COP samples (i.e., 2800 in this study).


(2) $$S\,a\,m\,p\,l\,e\,e\,n\,t\,r\,o\,p\,y\;\left(m,r,N\right)=-l\,n\,\frac{\emptyset^{m+1}\,\left(r\right)}{\emptyset^{m}\,\left(r\right)}$$

where *m* represents the embedding dimension (set to 2), *r* represents the tolerance for matching (set to 20% of the standard deviation of the filtered COP time series), *N* is the number of COP samples, and ∅ is the probability that vectors within a tolerance *r* of each other at length *m* will remain within tolerance *r* when the vector length is increased to *m+1* (Busa and van Emmerik, 2016).

### 2.5 Statistical analysis

Statistical analyses were performed using a combination of parametric and non-parametric tests, depending on the distribution of the data. Outcomes from three trials under congruent and incongruent conditions were pooled together for further statistical analysis to retain trial-by-trial variability. For self-reported anxiety scores, a paired-sample Wilcoxon signed-rank test was used to compare differences between the two congruent and incongruent conditions. For COP variables, normality was assessed using the Shapiro-Wilk test. If data were normally distributed, a paired *t*-test was used to compare the congruent and incongruent conditions. If data were non-normally distributed, a paired-sample Wilcoxon signed-rank test was used. The significance level was set at *p* < 0.05 for all tests. Effect sizes (Cohen’s d) were calculated for all significant *t*-tests to quantify the magnitude of the observed differences. Additionally, Pearson correlation coefficients were calculated to examine the relationships between the self-reported anxiety score and the COP variables for incongruent conditions. To account for multiple comparisons, a Bonferroni correction was applied, resulting in an adjusted significance level of *p* = 0.05/4 = 0.0125.

## 3 Results

### 3.1 Subjective anxiety score

Table 1 showed significantly higher self-reported anxiety levels during the incongruent task compared to the congruent task (Congruent: 2.22 ± 1.27, Incongruent: 2.50 ± 1.45, *p* = 0.02). This statistically significant increase in self-reported anxiety scores confirmed that the sensorimotor mismatch manipulation was successful in inducing self-reported anxiety within the experimental context.

**TABLE 1:** Paired differences for outcome measures (incongruent - congruent).

Outcomes	Mean of paired differences	SD of paired differences	*p*-value	Effect size
Self-reported anxiety	0.31	0.95	0.02	0.32
Total path length (mm)	24.93	120.01	0.14	
Movement area (mm^2^)	310.80	503.31	<0.001	0.62
Sample entropy in ML direction	−0.07	0.15	<0.001	0.47
Sample entropy in AP direction	−0.04	0.11	0.003	0.36

AP, anterior-posterior; ML, medial-lateral.

### 3.2 Voluntary postural control

Compared to the congruent task, Table 1 showed significantly larger COP movement area (Congruent: 235.20 ± 112.52 mm^2^, Incongruent: 546.06 ± 522.64 mm^2^, *p* < 0.001), lower sample entropy values for COP in AP direction (Congruent: 0.92 ± 0.15, Incongruent: 0.88 ± 0.18, p = 0.003) and COP in ML direction (Congruent: 1.20 ± 0.17, Incongruent: 1.13 ± 0.21, *p* < 0.001) during the incongruent task. However, there was no statistically significant difference in total length path of COP trajectory between congruent and incongruent tasks (Congruent: 645.61 ± 112.40 mm, Incongruent: 669.02 ± 122.51 mm, *p* = 0.14).

### 3.3 Relationship between anxiety score and voluntary control performance

**FIGURE 2:**
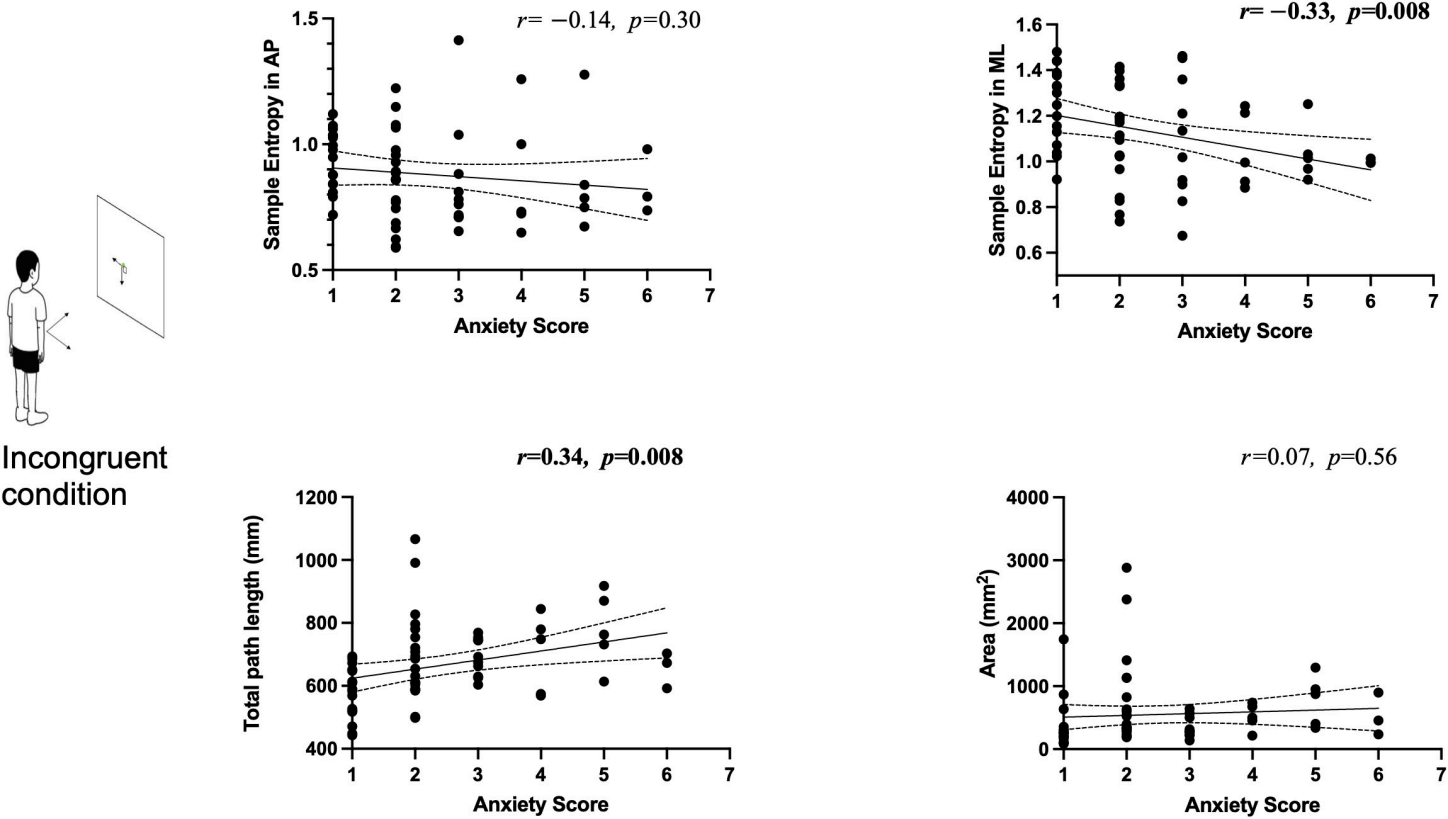
Relationship between self-reported anxiety scores and center of pressure (COP) measures during the incongruent condition. Significant correlations surviving the Bonferroni correction are highlighted in bold. An illustration of a person during the incongruent condition is shown on the left.

During the incongruent task, significant correlations were observed between anxiety scores and measures of voluntary postural control: (1) a significant negative correlation between anxiety score and COP sample entropy in the AP direction (*r* = –0.33, *p* = 0.008) and (2) a significant positive correlation between anxiety score and COP total path length (*r* = 0.34, *p* = 0.008) (Figure 2). However, no significant correlations were found between anxiety scores and other COP measures, i.e., COP sample entropy in the ML direction and COP area (Figure 2).

## 4 Discussion

This study investigated how sensorimotor mismatch affects voluntary postural control and the role of self-reported anxiety in modulating motor performance. Our findings demonstrate that sensorimotor mismatch reduces movement automaticity during goal-directed tasks, as evidenced by lower sample entropy values. Additionally, higher self-reported anxiety levels were associated with reduced automaticity of movement control in the ML direction under sensorimotor mismatch conditions.

### 4.1 Sensorimotor mismatch reduces automaticity of voluntary postural control

Previous research has primarily focused on movement trajectory errors caused by sensorimotor mismatch (Hadjiosif et al., 2021; Telgen et al., 2014). This study builds upon this prior work by applying sample entropy to quantify the impact of sensorimotor mismatch on movement automaticity. Our results showed significantly lower sample entropy values in both the AP and ML directions under the incongruent condition compared to the congruent condition (*p* < 0.01) (Table 1). These findings support our hypothesis that sensorimotor mismatch disrupts the automaticity of voluntary postural control.

Sensorimotor mismatch likely increases cognitive demands by requiring participants to consciously adjust their movements to align with altered visual feedback. This shift toward conscious control may involve increased activity in the dorsolateral prefrontal cortex (Fink et al., 1999). On the other hand, the goal-directed task was more challenging under the incongruent condition, which probably further decreased movement complexity and automaticity (Longo and Meulenbroek, 2018).

The forward internal model provides a theoretical framework for understanding these effects. This model posits that the brain predicts the sensory consequences of motor commands and adjusts motor output accordingly (Kawato, 1999). When sensory feedback is perturbed, such as under sensorimotor mismatch, these internal models must be updated to account for prediction errors. Neurophysiological evidence suggests that the cerebellum plays a key role in maintaining and updating these forward models (Ebner and Pasalar, 2008). Moreover, Klingner et al. (2022) showed that sensorimotor mismatch alters cerebellar connectivity, potentially disrupting its role in supporting automatic movements (Lang and Bastian, 2002). These findings align with our observation of reduced movement automaticity under sensorimotor mismatch conditions.

Our study included two COP measures to quantify the precision of voluntary movement, i.e., COP total path length and area, and found that the COP area was significantly larger under the incongruent condition, while the total path length did not differ significantly between conditions. This suggests the continuous goal-directed exploration of the center of mass under the incongruent condition, with a reduced ability to limit COP within a targeted area, indicating worse movement precision.

### 4.2 Relationship between self-reported anxiety and voluntary control under the sensorimotor mismatch

Our results revealed significantly higher self-reported anxiety levels during the sensorimotor mismatch condition compared to the congruent condition. According to the influential model of anxiety, the continuous mismatch between predicted and actual sensory events activates the behavioral inhibition system, which is a neural substrate closely associated with anxiety (McNaughton and Gray, 2000). As a result, participants probably increase self-perceived anxiety in response to the continuous sensorimotor conflicts.

The increased self-reported anxiety was associated with reduced ML movement automaticity (lower sample entropy in the ML direction) and larger total path length during the incongruent condition (Figure 2). These findings align with execution focus models, which posit that anxiety redirects attention inward, increasing conscious movement monitoring and disrupting automatic control processes (Beilock and Carr, 2001; Nieuwenhuys and Oudejans, 2012). Although we did not explicitly measure attentional focus, participants likely directed more attention internally to their own movements rather than externally toward the green cursor during the incongruent task, contributing to the observed reduction in sample entropy values.

We observed that the sensorimotor mismatch had a greater impact on the COP sample entropy in the ML direction compared to the AP direction, as revealed by higher paired differences and effect size in Table 1. Also, our correlation findings show that ML COP sample entropy was significantly associated with self-reported anxiety levels, rather than AP COP sample entropy (Figure 2). The differences in AP and ML directions may reflect different control strategies. In the AP direction, postural control is primarily relied on ankle strategies and resembles an inverted pendulum model. The ankle joint muscles generate torque to control forward and backward COP sway, and small ankle joint tilts can lead to noticeable COP displacements of several centimeters. In contrast, ML control relies more on shifts of body weight and often involves multi-joint strategies, particularly during voluntary goal-directed tasks. As voluntary movement in the ML direction increased more than that in the AP direction (Latash et al., 2003), maintaining the COP within the targeted area in the ML direction is more challenging, particularly under the incongruent condition.

While sensorimotor mismatch increased self-reported anxiety and reduced movement automaticity, this process shares similarities with error augmentation strategies that deliberately introduce perturbations to enhance learning (Sharp et al., 2011). Although higher self-reported anxiety correlated with decreased automaticity in our study, this shift from automatic to more conscious control might paradoxically enhance motor learning by increasing attention to task-relevant sensory cues–a mechanism similar to how error augmentation operates.

The Challenge Point Theory suggests that optimal learning occurs when task difficulty appropriately matches the learner’s skill level (Guadagnoli and Lee, 2004) and research shows that error amplification benefits skilled individuals more than novices (Marchal-Crespo et al., 2017). Similarly, the learning effects of sensorimotor mismatch-induced anxiety likely depend on individual skill levels and anxiety thresholds. Recent evidence by Cabral et al. (2024) demonstrates that practicing under mild anxiety during early learning phases improves performance under subsequent high-pressure conditions, suggesting that anxiety-induced attentional shifts may strengthen the encoding of motor programs despite initial performance costs.

### 4.3 Limitations

This study has several limitations that should be addressed in future research. First, we relied on self-reported measures of state anxiety using a Likert scale, which may be subject to bias or variability across participants. Future studies should consider incorporating objective physiological measures of anxiety, such as electrodermal activity or heart rate variability, to provide additional insights into the relationship between anxiety and motor performance during conditions of sensorimotor mismatch.

Second, while we successfully manipulated state anxiety through sensorimotor mismatch, we did not assess participants’ trait anxiety levels prior to the experiment, which may have influenced how individuals respond to sensorimotor mismatch or experience state anxiety during goal-directed tasks. Including trait anxiety assessments in future studies would help clarify its role in modulating motor performance.

## 5 Conclusion

This study demonstrated that sensorimotor mismatch reduces movement automaticity during goal-directed tasks. It also showed that higher anxiety levels were associated with reduced movement automaticity and decreased precision of control during tasks involving altered visual feedback. These findings advance our understanding of how anxiety and sensorimotor mismatch interact to affect motor control processes. From a clinical perspective, these results highlight the importance of assessing and managing anxiety when working with individuals with sensorimotor integration deficits. Disorders such as dystonia are characterized by impairments in sensorimotor integration, which can create mismatches between sensory inputs and motor outputs (Cuny et al., 2008; Lerner et al., 2004). Anxiety may exacerbate these deficits by further impairing automatic control processes essential for efficient motor execution.

## Data Availability

The raw data supporting the conclusions of this article will be made available by the authors, without undue reservation.
